# Modelling Radiation Cancer Treatment with a Death-Rate Term in Ordinary and Fractional Differential Equations

**DOI:** 10.1007/s11538-023-01139-2

**Published:** 2023-04-25

**Authors:** Nicole Wilson, Corina S. Drapaca, Heiko Enderling, Jimmy J. Caudell, Kathleen P. Wilkie

**Affiliations:** 1 Department of Mathematics, Toronto Metropolitan University, Toronto, Canada; 2 Engineering Science and Mechanics, Pennsylvania State University, University Park, USA; 3 Department of Integrated Mathematical Oncology, H. Lee Moffitt Cancer Center and Research Institute, Tampa, USA; 4 Department of Radiation Oncology, H. Lee Moffitt Cancer Center and Research Institute, Tampa, USA

**Keywords:** Mathematical oncology, Modelling $\&$ simulation, Cancer radiotherapy, Fractional derivatives, Differential equations

## Abstract

Fractional calculus has recently been applied to the mathematical modelling of tumour growth, but its use introduces complexities that may not be warranted. Mathematical modelling with differential equations is a standard approach to study and predict treatment outcomes for population-level and patient-specific responses. Here, we use patient data of radiation-treated tumours to discuss the benefits and limitations of introducing fractional derivatives into three standard models of tumour growth. The fractional derivative introduces a history-dependence into the growth function, which requires a continuous death-rate term for radiation treatment. This newly proposed radiation-induced death-rate term improves computational efficiency in both ordinary and fractional derivative models. This computational speed-up will benefit common simulation tasks such as model parameterization and the construction and running of virtual clinical trials.

## Introduction

Mathematical oncology aims to use mathematical and computational techniques to better understand cancer growth and progression and to better treat the disease. With the increased prevalence of mathematical and computational modelling in oncology, a shift has started from standard treatment protocols applied to everyone to more personalized treatment protocols developed for the patient (Baldock et al. 2013; Poleszczuk et al. 2018).

The desire to more accurately model patient response can lead to either more complex mathematical models, such as those employing fractional derivatives (Camargo et al. 2014; Arfan et al. 2021), or more general models with patient-specific parameterizations (Poleszczuk et al. 2018; Powathil et al. 2015; Rockne et al. 2010). Indeed, the need for patient-specific treatment plans is driven by the vastly different outcomes observed in clinical practice due to, among other factors, patient heterogeneity. Virtual clinical trials attempt to address this heterogeneity but can be computationally intensive.

It has been proven experimentally that cells behave as viscoelastic materials, for instance (Kasza et al. 2007). Furthermore, the viscoelastic response of a cell depends on its biological state (Nguyen et al. 2020). Thus, it is reasonable to assume that the cell dynamics due to biological processes correlates to its viscoelastic behaviour. The Caputo fractional derivative introduced in Caputo (1967) has been successfully used in constitutive laws of viscoelasticity for a long time, which were validated experimentally even in biological materials (Meral et al. 2010).

Riemann, Liouville, Cauchy, and Abel proposed various definitions for a fractional derivative with power-law kernel. Caputo modified the definition to incorporate an initial condition, making it appropriate for many real-world medical applications, such as models of hydrocephalus (Wilkie et al. 2011) and COVID-19 (Tuan et al. 2020). The Caputo definition is the most commonly applied definition in tumour growth models (Farayola et al. 2021). A new definition was proposed by Caputo and Fabrizio to use a smooth exponential kernel (Caputo and Fabrizio 2015) and remove the singularity. This definition is claimed to better describe material heterogeneities and fluctuations on different scales. Atangana-Baleanu later proposed non-local and non-singular kernel definitions based on the Caputo or Riemann–Liouville approach, using the Mittag–Leffler function (Atangana and Baleanu 2016). However, it appears that all these non-singular kernel definitions do not have convolution representations, they require restrictive assumptions on the initial condition, and their corresponding modified forms simplify to representations involving classical and Caputo derivatives (Diethelm et al. 2020). Consequently, in this work, the more commonly used Caputo fractional derivative will be used.

Fractional derivatives have been applied successfully to many different applications where the material properties exhibit heterogeneities and multiscale fluctuations with memory effects (Ionescu et al. 2017), including cancer modelling. A fractional Gompertz model was discussed in Bolton et al. (2015), wherein they showed a slight improvement in fit to data over the standard model. In Valentim et al. (2020), several standard tumour growth models were compared in both ordinary and fractional differential equation forms. They showed that the fractional order may be advantageous as it significantly affects the predicted growth dynamics. Arik and Araz presented a piecewise model for cancer radiotherapy wherein they used an ordinary derivative model in the pre-treatment phase, a stochastic model in the treatment phase, and a fractional model in the post-treatment phase (Arık and İğret Araz 2022). In Solís-Pérez et al. (2019), both Caputo and Caputo–Fabrizio definitions of the fractional derivative were applied to a breast cancer model. They claim the memory effect was more prolonged in the Caputo–Fabrizio model. And in Morales-Delgado et al. (2019), the Caputo–Fabrizio and Atangana–Baleanu definitions were used in a model of cancer chemotherapy, wherein the time history captured new dynamics of the chemotherapy treatment. Additionally, optimal control has been explored in fractional cancer treatment models (Akman Yıldız et al. 2018; Sweilam et al. 2020; Baleanu et al. 2019).

Perhaps the most significant use of tumour growth and treatment mathematical models is the suggestion and testing of new treatment strategies. Mathematical modelling in the field of oncology allows for inexpensive, risk-free exploration to be done where experimental testing is not possible or carries significant costs. It should be noted that in a clinical setting experimental testing of many treatment schedules is neither feasible nor ethical, and tumour measurement data are limited at best. The challenge then is to develop models, which are complex enough to adequately describe the tumour growth, but simple enough to give insight that can be used clinically for predictive purposes. To this end, models should be calibrated and validated on separate datasets and then evaluated for predictive power before being used to explore alternatives (Brady and Enderling 2019). Limited clinical data make determining the numerous model parameters of highly complex models impossible, and so the aim must be to minimize the number of model parameters in order to avoid model over-fitting and uncertainty.

To this end, we use clinical data of 19 head and neck cancer patients (Caudell et al. 2021) to compare 6 mathematical models: three standard ordinary differential equation-based models and the three related fractional differential equation-based models. We fit each model to each patient and explore the resulting parameter landscape. We also quantify the goodness of fit of these 6 models using several information criterion in order to determine the best model for the clinical data.

In this work, we use the Caputo fractional derivative, which, for uniqueness of solutions, requires a continuous growth expression (Diethelm et al. 2002). This motivated the development of a continuous death-rate term to describe the effects of radiation treatment. The new radiation effect model is compared to both a discontinuous expression, and the standard impulsive model (Alvord et al. 2008; Enderling et al. 2010) which requires the stopping and starting of the numeric integration at each treatment time. In addition to being a more biological representation of radiation treatment effects, we found that the new death-rate model provided a considerable increase in computation speed (about twice as fast), which will provide significant benefit to large computational tasks such as model parameterizations and virtual clinical trials.

## Model Development

We consider three standard models of tumour growth: exponential growth (1), logistic growth (2), and exponential-linear growth (3):

1 $$\begin{aligned} \frac{dv}{dt}&= a v \end{aligned}$$

2 $$\begin{aligned} \frac{dv}{dt}&= a v \left( 1 - \frac{v}{K} \right) \end{aligned}$$

3 $$\begin{aligned} \frac{dv}{dt}&= {\lambda _0 v} {\left( 1 + \left( \frac{\lambda _0}{\lambda _1} v \right) ^\psi \right) ^{-\frac{1}{\psi }}} \end{aligned}$$

In all equations, *v*(*t*) is the tumour volume in cm^3^, and we assume that time has been non-dimensionalized using a characteristic time scale of 1 day. In Eq. (1), *a* is the (dimensionless) exponential growth rate. In Eq. (2), *a* is the (dimensionless) intrinsic growth rate and *K* is the carrying capacity in cm^3^. And in Eq. (3), $\lambda _0$ is the (dimensionless) exponential growth rate, $\lambda _1$ is the linear growth rate (with dimensions cm^3^), and $\psi$ is a transition factor taken to be $\psi =20$ to ensure a sufficiently smooth transition between the exponential and linear growth phases.

### A Continuous Radiation Death Rate

To incorporate the effect of radiation treatment, we use the standard linear-quadratic (LQ) model (McMahon 2019), which predicts that following a dose of *d* Gy, the surviving fraction of a population is given by $SF = \exp (-\alpha d - \beta d^2)$, where $\alpha$ (Gy^-1^) and $\beta$ (Gy^-2^) describe the tissue radio-sensitivities. Typically, in mathematical models, radiation is assumed to cause an instantaneous cell kill equal to the proportion predicted by the LQ model, $v(1-SF)$. This amount of cell death is instantaneously removed from the simulated tumour (Prokopiou et al. 2015; Holdsworth et al. 2012) by stopping the numerical integration at treatment time $\tau _i$ and restarting it again with an updated tumour volume of $v(\tau _i) SF$. This method of stopping and starting the numerical integration should not be used with fractional derivatives because they require continuity to guarantee existence and uniqueness of solutions (Diethelm et al. 2002). Further, the fractional differential operator incorporates a history-dependence into the growth dynamics, which would be lost each time the numeric integration is stopped. To avoid these issues, we propose a continuous radiation death-rate term instead of the standard instantaneous death impulse train to model radiation treatment.

We proceed as follows. For each dose fraction *d* delivered over (dimensionless) time window *tw*, we propose that the death rate can be described as:

$$\begin{aligned} \text {death rate} = v\left( 1 - SF \right) \frac{1}{tw} = v \left( 1 - e^{-\alpha d - \beta d^2} \right) \frac{1}{tw}. \end{aligned}$$

This death rate is in effect for the treatment window length, *tw*, starting at treatment time $\tau _i$, otherwise it is zero as there is no death assumed between radiation treatments. We can create a train of all treatments in the therapy protocol by summing over all treatment days $\tau _i \in {\mathcal {T}}$ and using a continuous approximation to the Heaviside function to switch the treatment on and off while maintaining continuity of our death-rate function:

$$\begin{aligned} \text {death rate} = \sum _{{\mathcal {T}}} v(t) \left( 1 - e^{-\alpha d -\beta d^2} \right) \frac{ {\hat{H}}(t-\tau _i) - {\hat{H}}(t - (\tau _i+tw)) }{tw}. \end{aligned}$$

where ${\hat{H}}(t)$ is a continuous approximation of the Heaviside function defined in terms of hyperbolic tangents (Bylsma 2012):

$$\begin{aligned} {\hat{H}}(t) = \frac{1}{2} \left( 1 + \tanh \Big (\frac{t}{\epsilon }\Big )\right) , \end{aligned}$$

with $0 < \epsilon \ll 1$ to capture the steep jump of the Heaviside function. Here, we use $\epsilon = 10^{-5}$.

Adding this new death-rate term into our tumour growth equations gives three ODE models for tumour growth with radiation therapy:

4 $$\begin{aligned} \frac{dv}{dt}&= a v - v\left( 1 - e^{-\alpha d -\beta d^2} \right) \sum _{{\mathcal {T}}} \frac{{\hat{H}}(t-\tau _i) - {\hat{H}}(t-\tau _i-tw)}{tw} \end{aligned}$$

5 $$\begin{aligned} \frac{dv}{dt}&= a v \left( 1 - \frac{v}{K} \right) - v\left( 1 - e^{-\alpha d -\beta d^2} \right) \sum _{{\mathcal {T}}} \frac{{\hat{H}}(t-\tau _i) - {\hat{H}}(t-\tau _i-tw)}{tw} \end{aligned}$$

6 $$\begin{aligned} \frac{dv}{dt}&= {\lambda _0 v} {\left( 1 + \left( \frac{\lambda _0}{\lambda _1} v \right) ^\psi \right) ^{-\frac{1}{\psi }}} - v\left( 1 - e^{-\alpha d -\beta d^2} \right) \sum _{{\mathcal {T}}} \frac{{\hat{H}}(t-\tau _i) - {\hat{H}}(t-\tau _i-tw)}{tw}. \end{aligned}$$

Notice that in the limit as $tw \rightarrow 0$, the treatment window function $\frac{ {\hat{H}}(t-\tau _i) - {\hat{H}}(t-\tau _i-tw) }{tw} \rightarrow \delta (t-\tau _i)$ where $\delta (t)$ is the Dirac-delta function. As the treatment window shrinks, the death rate increases to affect the same total death as predicted by the LQ model. Thus, in the limit as the treatment window shrinks to zero, our continuous death-rate term approaches the standard impulsive model.

### Fractional Derivative Tumour-Radiation Models

Finally, we can convert our time-dimensionless ODE models of tumour growth and radiation into fractional differential equations (FDE) by replacing the left-hand side of Eqs. (4)–(6) with the Caputo fractional derivative $D^\mu v$. The Caputo fractional derivative, assuming that time starts at $t=0$ and that the fractional order $\mu$ lies in $0<\mu \le 1$, is defined by:

$$\begin{aligned} D^{\mu }f(t)=\frac{1}{\Gamma (1-\mu )} \int _0^t \frac{1}{(t-\xi )^{\mu }} \frac{df}{d\xi } \,d\xi , \end{aligned}$$

where $\Gamma (x)$ is the Gamma function. When $\mu = 1$, the ordinary derivative of first order is obtained, that is $D^1 f = \frac{df}{dt}$.

In Eqs. (4)–(6), replacing the ordinary derivative with the Caputo fractional derivative of order $\mu$ gives the following three FDE models for tumour growth with radiation treatment:

7 $$\begin{aligned} D^\mu v&= a v - v\left( 1 - e^{-\alpha d -\beta d^2} \right) \sum _{{\mathcal {T}}} \frac{{\hat{H}}(t-\tau _i) - {\hat{H}}(t-\tau _i-tw)}{tw} \end{aligned}$$

8 $$\begin{aligned} D^\mu v&= a v \left( 1 - \frac{v}{K} \right) - v\left( 1 - e^{-\alpha d -\beta d^2} \right) \sum _{{\mathcal {T}}} \frac{{\hat{H}}(t-\tau _i) - {\hat{H}}(t-\tau _i-tw)}{tw} \end{aligned}$$

9 $$\begin{aligned} D^\mu v&= {\lambda _0 v} {\left( 1 + \left( \frac{\lambda _0}{\lambda _1} v \right) ^\psi \right) ^{-\frac{1}{\psi }}} - v\left( 1 - e^{-\alpha d -\beta d^2} \right) \sum _{{\mathcal {T}}} \frac{{\hat{H}}(t-\tau _i) - {\hat{H}}(t-\tau _i-tw)}{tw}. \end{aligned}$$

These FDE models contain one more parameter than the corresponding ODE models, the order of the fractional derivative $\mu$, assumed to lie in (0, 1].

### Model Parameterization

To numerically simulate both ODE and FDE models, we use the same solver for consistency, namely the FDE solver *fde12* in MATLAB (Garrappa 2012), which implements a predictor–corrector algorithm (Diethelm et al. 2002). To reduce the dimensionality of our parameterization, we make the following assumptions. Unless otherwise stated, we assume that all treatment windows, *tw*, are 15 minutes (non-dimensionalized). We assume that $\frac{\alpha }{\beta }= 10$ Gy as suggested by clinical literature for head and neck cancers (Bel et al. 2018). Thus, we fit $\alpha$ and determine $\beta$ from $\beta = \frac{\alpha }{10}$ Gy^-2^. Lastly, we fix the carrying capacity *K* (cm^3^) of the logistic model to be twice the initial volume measurement for that patient. This assumption enforces a logistic slow-down in the growth curve, further differentiating it from the exponential growth model. With these assumptions, it remains to determine parameters *a* and $\alpha$ for the exponential and logistic models, and parameters $\lambda _0$, $\lambda _1$, and $\alpha$ for the exponential-linear model. For the corresponding fractional derivative models, we must also determine the fractional order $\mu$.

To parameterize our models, we use clinical data from 19 head and neck cancer patients treated at the Moffitt Cancer Center and previously published in Caudell et al. (2021). The patients received between 66–70 Gy of radiation in 2 Gy weekday fractions in addition to chemotherapy. As done in Caudell et al. (2021), we neglect the effect of the chemotherapy and assume the tumour response is due to the radiation treatment alone. This assumption causes the radio-sensitivities of our fits to be over-estimates as they also incorporate any tumour reduction due to chemotherapy.

Tumour volume measurements were taken prior to treatment start for planning purposes, just before the first radiation fraction, and then weekly for the remainder of treatment. The final measurement is assumed to occur on the last day of treatment. Each patient *j* has a specific set of treatment days ${\mathcal {T}}_j$ for $j = 1 \ldots 19$, with a subset of ${\mathcal {T}}_j$ corresponding to measurement days.

Unknown model parameters were fit using the gradient-based *fmincon* in MATLAB with generous but physiological upper and lower bounds imposed: *a*, $\lambda _0$, $\lambda _1$, and $\mu$ must lie in (0.001, 1), and $\alpha$ must lie in $(0,\infty )$. Best-fit parameters where those that minimized the sum of squared residuals (SSR) between the numerical solution and the patient’s data, over several repetitions of the fitting process. Initial guesses for the optimization scheme were uniformly sampled random numbers in the range (0, 0.1) for parameters *a*, $\alpha$ (Gy^-1^), and $\lambda _0$, and in the range (0, 1) for parameter $\lambda _1$ (cm^3^). The initial guess of the fractional order was $\mu = 0.5$ to start the fitting algorithm in the middle of our accepted range of (0, 1].

## Results

### Fitted Parameter Values

**Fig. 1:**
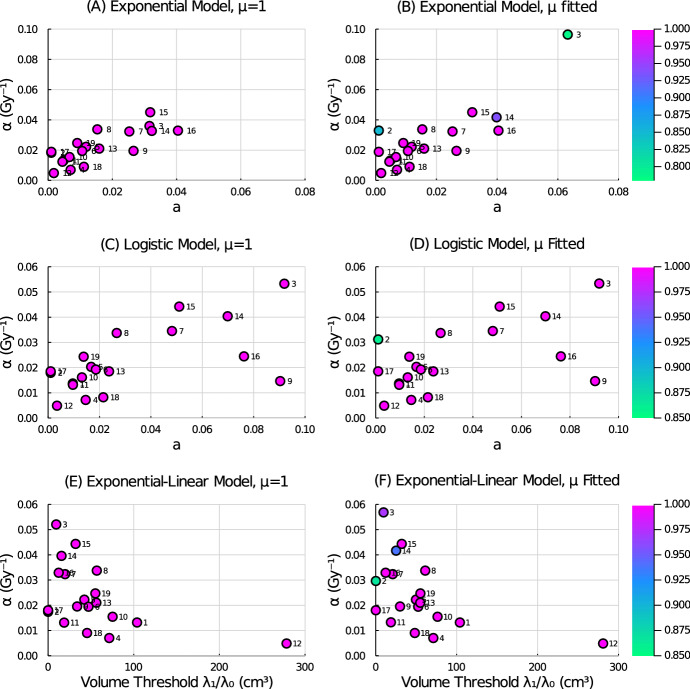
Parameter landscapes for each ODE and FDE model. ODE models are shown in **A** exponential model, **C** logistic model, and **E** exponential-linear model. FDE models are shown in **B** fractional exponential model, **D** fractional logistic model, and **F** fractional exponential-linear model. Color indicates the order of the derivative with magenta corresponding to an order-one derivative. Patient number is indicated beside the corresponding data point

**Fig. 2:**
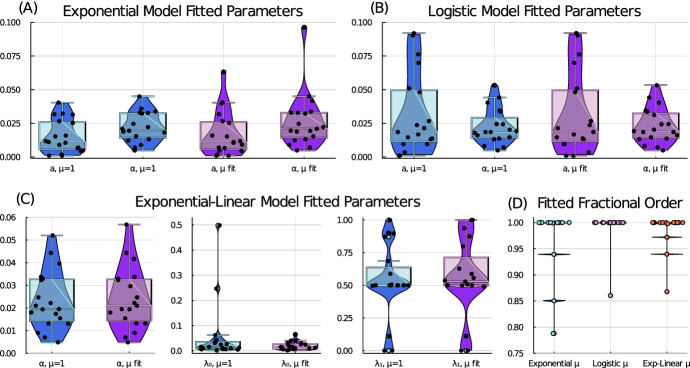
Violin box plots for all fitted model parameter values for each ODE and FDE model: **A** exponential model, **B** logistic model, **C** exponential-linear model, and **D** the fractional order for each model

A summary of the parameter fits for our six tumour growth models with radiation treatment (Eqs. (4)–(9)) to the 19 patient datasets is shown in Table 1. From the parameter landscapes shown in Fig. 1, the spread of the patient-specific parameter values is clearly seen, as well as the small deviations in fits caused by the introduction of a fractional-ordered derivative. Violin box plots for all parameter values for each model show the variability across the patient cohort, see Fig. 2.

**Table 1:** Key statistical measures of fitted model parameters for all models and, as an example, the fitted parameter values for Patient 5

Model and Parameter	Min	Max	Mean	SD	Median	Q1	Q3	Patient 5
Exponential (4)								
*a*	0.0010	0.0404	0.0152	0.0123	0.0111	0.0053	0.0263	0.0118
$\alpha$ (Gy^-1^)	0.0048	0.0450	0.0220	0.0109	0.0195	0.0137	0.0326	0.0222
Fractional exponential (7)								
*a*	0.0010	0.0632	0.0173	0.0167	0.0111	0.0053	0.0263	0.0118
$\alpha$ (Gy^-1^)	0.0048	0.0964	0.0265	0.0204	0.0210	0.0137	0.0329	0.0222
$\mu$	0.7880	1.0000	0.9777	0.0585	1.0000	0.999998	1.0000	1.0000 (0.9999999835)
Logistic (5)								
*a*	0.0010	0.0919	0.0316	0.0301	0.0186	0.0105	0.0503	0.0168
$\alpha$ (Gy^-1^)	0.0049	0.0533	0.0225	0.0131	0.0185	0.0139	0.0314	0.0203
Fractional logistic (8)								
*a*	0.0010	0.0920	0.0316	0.0302	0.0186	0.0105	0.0503	0.0168
$\alpha$ (Gy^-1^)	0.0049	0.0534	0.0232	0.0132	0.0192	0.0139	0.0331	0.0203
$\mu$	0.8605	1.0000	0.9926	0.03199	1.0000	0.999998	1.0000	1.0000 (0.9999999365)
Exponential linear (6)								
$\lambda _{0}$	0.0018	0.4985	0.0566	0.1201	0.0153	0.0075	0.0381	0.0118
$\lambda _{1}$ (cm^3^)	0.0010	1.0000	0.5296	0.2770	0.5007	0.5004	0.6614	0.5007
$\alpha$ (Gy^-1^)	0.0048	0.0521	0.0231	0.0129	0.0195	0.0137	0.0328	0.0222
Fractional exponential linear (9)								
$\lambda _{0}$	0.0018	0.0653	0.0190	0.0160	0.0118	0.0075	0.0263	0.0118
$\lambda _{1}$ (cm^3^)	0.0010	1.0000	0.5558	0.2924	0.5287	0.4990	0.7553	0.5874
$\alpha$ (Gy^-1^)	0.0048	0.0568	0.0242	0.0137	0.0210	0.0138	0.0328	0.0222
$\mu$	0.8678	1.0000	0.9883	0.0328	1.0000	0.999980	1.0000	1.0000 (0.9999999969)

Overall, between the ODE and corresponding FDE model, the parameter fits are mostly similar. The majority of patients have fits requiring the order of the fractional derivative $\mu$ to satisfy $0.999 \le \mu \le 1$. At least 15 out of the 19 patients, for all FDE models, had $\mu$ fits that satisfied this range. This suggests that the fractional derivative is not being employed to moderate the tumour response curve and that an ordinary differential operator would result in a similar fit with the added benefits of reducing the model complexity, reducing the number of model parameters, and returning the numeric simulation to more standard methods.

**Fig. 3:**
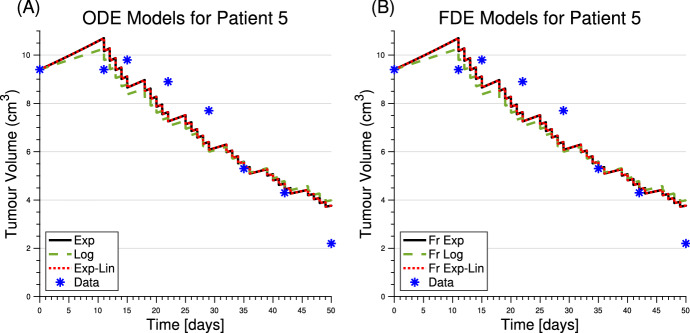
Model simulations and patient data for patient 5. All ODE models are shown in **A**, and all FDE models are shown in **B**. Parameter values are listed in Table 1

**Fig. 4:**
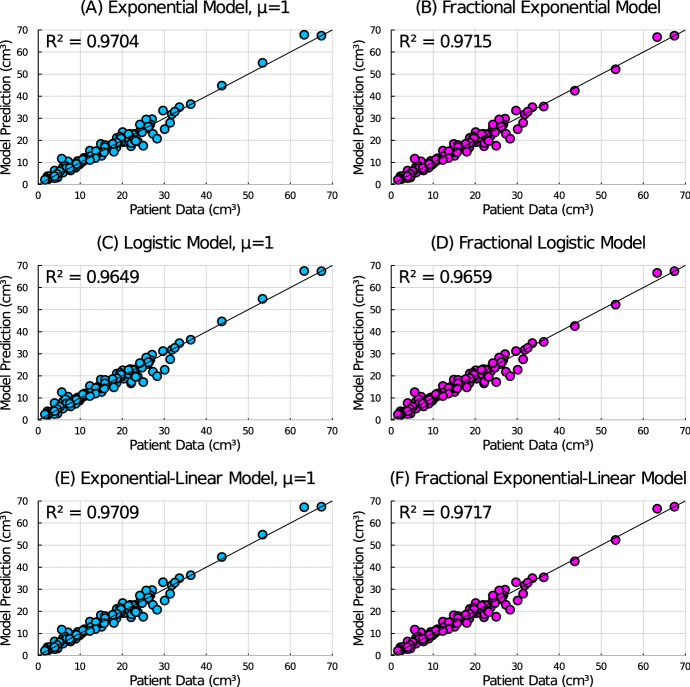
Predicted tumour volume vs actual tumour volume for all patients using the **A** exponential model, **B** fractional exponential model, **C** logistic model, **D** fractional logistic model, **E** exponential-linear model, and **F** fractional exponential-linear model

To demonstrate the data and model fits, the ODE and FDE model predictions are shown for patient 5 in Fig. 3. Patient 5 was selected to demonstrate a typical result since the fitting results for this patient resulted in the median SSR values over all patients. The relationships between model predictions and patient data, for all patients and each model, are shown in Fig. 4.

### Dynamic Sensitivity Analysis

To examine the effects of fitted model parameter values on the predicted tumour response, we use a dynamic method of local sensitivity analysis. We examine the time-response of relative sensitivity coefficients, ${\bar{S}}_{p_j}^v(t)$, which measure the relative change in predicted tumour volume, *v*, to the relative change in the perturbed model parameter $p_j$. In other words, the standard sensitivity coefficients are normalized by the ratio of the perturbed parameter’s nominal value, $p_{j,\emptyset }$, to the nominal tumour volume at time *t*, $v_{\emptyset }(t)$. The relative sensitivity coefficients are defined by:

10 $$\begin{aligned} {\bar{S}}^v_{p_j}(t) = \frac{\partial v}{\partial p_j} \frac{p_{j,\emptyset }}{v_{\emptyset }} \approx \frac{v(t;p_j+\Delta p_j) - v(t;p_j)}{v_{\emptyset }(t)}\frac{p_{j,\emptyset }}{\Delta p_j}. \end{aligned}$$

where the subscript $\emptyset$ denotes the nominal (unperturbed) value of either the parameter or the predicted volume. Here we take $\Delta p_j = 0.01 p_{j,\emptyset }$ to reflect a 1% change in the parameter’s fitted value.

**Fig. 5:**
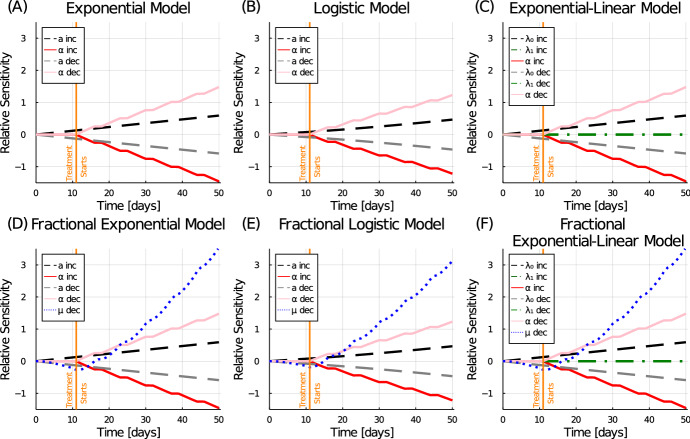
Relative sensitivity coefficients for all fitted model parameters of each model. Equation (10) is used to compute the relative sensitivity coefficients over times [0, 50]. Each parameter was perturbed independently by $1\%$ of the nominal value

We use the patient 5 data to explore the model parameter sensitivities. Relative sensitivities were calculated for all fitted parameters of each model from day 0–50, for both 1% increases and decreases in the nominal value. Since fractional order of patient 5 was very close to 1, we consider only a 1% decrease in the value. Figure 5 shows the resulting dynamics of the relative sensitivity coefficients for each model and its fitted parameters.

Note that across all models, the behaviour of the radiation sensitivity, $\alpha$, is similar. As expected, an increase to the radiation sensitivity parameter results in a reduction of tumour volume and thus a negative relative sensitivity. The sensitivity of parameter $\alpha$ grows in magnitude with each additional treatment. The exponential growth rate, *a* or $\lambda _0$, also has similar sensitivity behaviour across the models. A small increase to the exponential growth rate results in an increase to the tumour volume and thus a positive relative sensitivity. The sensitivity of *a* grows in magnitude as the difference in perturbed tumour volume to the nominal volume continues to grow exponentially. Note that for patient 5, the exponential-linear model threshold for transition from exponential to linear growth occurs at $\frac{\lambda _1 }{\lambda _0} > 40$ cm^3^. Since the tumour never grows this large under the prescribed treatment (see Fig. 3), this model is always in the exponential growth phase. Thus, the relative sensitivity of $\lambda _0$ is similar to that of exponential growth parameter *a*, and the relative sensitivity of the linear growth rate, $\lambda _1$, is negligible.

**Fig. 6:**
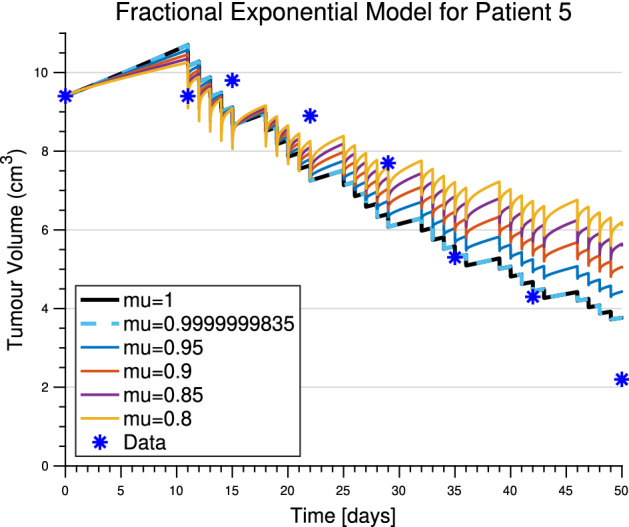
Fractional exponential model predictions for patient 5 with decreasing fractional order from $\mu =1$ to $\mu =0.8$

Perhaps the most interesting sensitivity behaviour comes from the fractional order $\mu$. With a small decrease in $\mu$, there is an initial decrease in tumour volume and thus relative sensitivity. This is followed by a sharp, and ever growing, increase once treatment begins. To explore this phenomenon further, treatment curves for patient 5 are simulated with decreasing values of fractional order, see Fig. 6. Initially the lower fractional ordered derivative has a slower growth rate, but once treatment starts, the lower orders exhibit faster regrowth rates post-treatment, ultimately leading to larger tumour volumes. In fact, the physicality of these accelerated regrowth rates post-treatment is questionable: see, for example, the $\mu =0.8$ curve in Fig. 6 which has near-infinite growth rates right after treatment application (vertical dips in the curve). This suggests that fractional derivative-based models are not a good fit for treatment prediction.

### Model Comparison

The Akaike information criterion (*AIC*) and Bayesian information criterion (*BIC*) are two tools that can be used to help identify the best model for a dataset, given the number of model parameters. Both metrics assume that the errors are normally distributed. This assumption was verified using both the Lilliefors and the Shapiro–Wilk tests, implemented in MATLAB. The model and patient combinations that have errors that failed the normality tests are: patient 10 with the exponential, fractional exponential, exponential-linear, and fractional exponential-linear models, and patient 19 with the logistic and fractional logistic models. The results for these patient-model combinations should be interpreted with caution, but are included below for completeness.

The Bayesian information criterion, BIC, is defined by:

$$\begin{aligned} \text {BIC} = n \ln \left( \frac{\text {SSR}}{n} \right) + {\mathcal {K}} \ln n, \end{aligned}$$

where *n* is the number of data points, ${\mathcal {K}}$ is the number of fitted model parameters, and SSR is the sum of squared errors between the data and model prediction. Since the patient data sets are small, we use the corrected Akaike information criterion (Brewer et al. 2016), AICc, defined by

$$\begin{aligned} \text {AICc} = n \ln \left( \frac{\text {SSR}}{n} \right) + 2 {\mathcal {K}} + \frac{2{\mathcal {K}}({\mathcal {K}}+1)}{n-{\mathcal {K}}-1} \end{aligned}$$

To aid in the comparison of these results, we normalize the AICc scores to the minimum score found for that dataset:

$$\begin{aligned} \Delta _i = \text {AICc}_i - \text {AICc}_{\text {min}}, \end{aligned}$$

where $\text {AICc}_i$ is the score for model *i*, and $\text {AICc}_{\text {min}}$ is the minimum score for all models applied to that dataset. AICc $\Delta _i$ values for all patients and models are reported in Table 2.

**Table 2:** AICc $\Delta _{i}$values for all patient-model combinations. $\Delta _i=0$ indicates the best candidate model for that patient. $\Delta _i\le 2$ indicates that substantial support exists in the data for that model (cells in italics). Increasing $\Delta _i$ values suggest decreasing support for that model. Models with moderate support, $2 < \Delta _i \le 7$, are in bold, and models with the minimal support, $\Delta _i > 7$, are in bolditalics

	Exp	Fr Exp	Log	Fr Log	Exp-Lin	Fr Exp-Lin
Patient 1	0	**5**.**60**	*1.43*	***7.03***	**5**.**60**	***14.93***
Patient 2	*0.84*	**2**.**03**	0	*1.70*	**4**.**98**	***10.66***
Patient 3	**3**.**79**	**3**.**95**	0	**5**.**60**	*1.73*	***10.99***
Patient 4	0	**7**.**00**	*0.83*	***7.83***	**7**.**00**	***21.00***
Patient 5	0	**5**.**60**	*1.33*	**6**.**93**	**5**.**60**	***14.93***
Patient 6	0	**5**.**60**	*1.86*	***7.46***	**5**.**60**	***14.93***
Patient 7	0	**5**.**60**	**4**.**42**	***10.02***	**5**.**60**	***14.93***
Patient 8	0	**5**.**60**	*1.54*	***7.14***	**5**.**60**	***14.93***
Patient 9	0	**7**.**00**	**4**.**41**	***11.41***	**7**.**00**	***21.00***
Patient 10	0	**4**.**80**	**2**.**07**	**6**.**87**	**4**.**80**	***12.00***
Patient 11	0	**5**.**60**	*0.15*	**5**.**75**	**5**.**51**	***14.84***
Patient 12	0	**5**.**60**	*0.37*	**5**.**97**	**5**.**60**	***14.93***
Patient 13	0	**5**.**60**	*0.76*	**6**.**36**	**5**.**60**	***14.93***
Patient 14	*0.50*	0	***14.07***	***19.67***	**2**.**82**	***9.33***
Patient 15	0	**5**.**60**	**3**.**91**	***9.51***	**5**.**65**	***14.98***
Patient 16	0	**5**.**60**	**2**.**12**	***7.72***	**5**.**60**	***14.93***
Patient 17	*0.14*	**5**.**74**	0	**5**.**60**	**5**.**46**	***14.80***
Patient 18	0	**5**.**60**	*0.54*	**6**.**14**	**5**.**60**	***14.93***
Patient 19	0	**4**.**80**	*1.98*	**6**.**78**	**4**.**80**	***12.00***

The AICc $\Delta _i$ values can be used to rank the six candidate models from best ($\Delta _i=0$) to worst (maximum $\Delta _i$) for each patient. If a tie occurs, both models are ranked at the average, for example if two models tie for third and fourth place, their assigned ranking would both be 3.5. For patient 5, the model ranking is: 1—exponential, 2—logistic, 3.5—fractional exponential, 3.5—exponential-linear, 5—fractional logistic, and 6—fractional exponential-linear. From the values listed in Table 2, for 15 of the 19 patients, the $\Delta _i$ values rank the exponential model as the best candidate model. The logistic model is ranked best for 3 of the 19 patients, and the fractional exponential model is ranked best for one patient.

While the AICc differences in Table 2 are useful for creating a ranking of the models, it is possible to extend our use of the $\Delta _{i}$-values to quantify the plausibility of each model being the best model from our set in the Kullback–Leibler sense (Anderson and Burnham 2002). We first calculate the likelihood of each model given the data as

$$\begin{aligned} {\mathcal {L}} (g_{i} \vert \text {data} ) \propto e^{-\frac{1}{2}\Delta _{i}}, \end{aligned}$$

where $g_{i}$ is the $i^{th}$ candidate model. These likelihoods represent the relative strength of evidence for each model. To better interpret the relative likelihood of the model, given the data and *R* different models, we normalize the ${\mathcal {L}} (g_{i} \vert \text {data})$ to be a positive set of Akaike weights (Anderson and Burnham 2002), $w_{i}$, defined by

11 $$\begin{aligned} w_{i}=\frac{e^{-\frac{1}{2}\Delta _{i}}}{\sum \limits _{r=1}^{R}e^{-\frac{1}{2}\Delta _{r}}}. \end{aligned}$$

Observe that $\sum \limits _{i=1}^{R}w_{i}=1$. Akaike weights are considered to be the weight of evidence in favour of model *i* as being the best model for a given dataset out of the *R* candidate models (Anderson and Burnham 2002). The weights for all patient-model combinations are listed in Table 3. Consideration of these weights shows that for all but four patients, namely patients 2, 3, 14, and 17, the exponential model has the highest probability and is thus considered the best of our six candidate models for the given data. Observe that for these same 15 patients, the logistic model, with fixed carrying capacity $K$, has the second highest probability. For patients 2, 3, and 17, the logistic model has the highest probability of being the best model.

**Table 3:** AICc weights, $w_{i}$, for all patient-model combinations. The largest weight in a row, in bold, indicates that there is the most evidence in favour of that model being the best over all other considered models, for that patient

	Exp	Fr Exp	Log	Fr Log	Exp-Lin	Fr Exp-Lin
Patient 1	**0**.**60910**	0.03704	0.29833	0.01814	0.03704	0.00035
Patient 2	0.25938	0.14302	**0**.**39448**	0.16851	0.03269	0.00191
Patient 3	0.08471	0.07833	**0**.**56355**	0.03422	0.23687	0.00232
Patient 4	**0**.**57459**	0.01735	0.37924	0.01145	0.01735	0.00002
Patient 5	**0**.**60002**	0.03649	0.30793	0.01873	0.03649	0.00034
Patient 6	**0**.**64899**	0.03946	0.25614	0.01558	0.03946	0.00037
Patient 7	**0**.**80729**	0.04909	0.08868	0.00539	0.04909	0.00046
Patient 8	**0**.**61960**	0.03768	0.28723	0.01747	0.03768	0.00035
Patient 9	**0**.**85185**	0.02572	0.09385	0.00283	0.02572	0.00002
Patient 10	**0**.**63604**	0.05770	0.22644	0.02054	0.05770	0.00158
Patient 11	**0**.**47388**	0.02882	0.44009	0.02676	0.03017	0.00028
Patient 12	**0**.**49908**	0.03035	0.41472	0.02522	0.03035	0.00029
Patient 13	**0**.**54106**	0.03290	0.37031	0.02252	0.03290	0.00031
Patient 14	0.38322	**0**.**49170**	0.00043	0.00003	0.12000	0.00462
Patient 15	**0**.**78681**	0.04785	0.11139	0.00677	0.04674	0.00044
Patient 16	**0**.**67091**	0.04080	0.23294	0.01417	0.04080	0.00038
Patient 17	0.44064	0.02680	**0**.**47276**	0.02875	0.03078	0.00029
Patient 18	**0**.**51718**	0.03145	0.39557	0.02405	0.03145	0.00030
Patient 19	**0**.**62920**	0.05708	0.23387	0.02122	0.05708	0.00156

In a similar manner as above, the BIC $\Delta _i$-values were calculated and used to rank the models for each patient. Summing the ranking over all patients for a particular model gives a tally score, indicating the overall ranking of the model in our 19-patient dataset. Table 4 provides a summary of the AICc and BIC analyses, including an overall ranking of the models from both measures. The exponential growth model was found to be the best model for the clinical dataset, with the logistic model (with fixed carrying capacity) coming in second. Of note, the FDE models ranked lower than their respective ODE models, indicating that the inclusion of a fractional derivative does not perform sufficiently better to overcome the penalty for including another fitted parameter.

**Table 4:** Summary of model comparison analysis. ${\mathcal {K}}$ is the number of parameters that were fit in the model. The column labelled ‘$p<0.05$’ indicates the fraction of patients for which the null hypothesis was rejected based on the Lilliefors and Shapiro–Wilk normality tests. The tally columns indicate the sum over all patients of the AICc or BIC $\Delta _i$-value rankings for that model

Rank	Model	${\mathcal {K}}$	Mean SSR	$p<0.05$	Mean AICc $\Delta _i$	AICc Tally	Mean $w_i$	Mean BIC $\Delta _i$	BIC Tally
1	Exp	2	25.83	1/19	0.28	24	0.56	0.65	32
2	Log	2	30.31	1/19	2.20	38	0.29	2.57	52
3	Fr Exp	3	24.65	1/19	5.10	66	0.07	1.89	60.5
4	Exp-Lin	3	25.25	1/19	5.27	66	0.05	2.06	61.5
5	Fr Log	3	29.43	1/19	7.66	93	0.03	4.45	95
6	Fr Exp-Lin	4	24.47	1/19	14.53	112	0.00	3.79	98

### Convergence of Radiation Effect to Impulsive Model

As the exponential model was found above to be the best candidate model for the clinical dataset, we proceed with the rest of our analysis using only this model. In Sect. 2.1, we discussed how the newly proposed continuous death-rate term introduced to model the effect of radiation-induced cell death approaches the impulsive model more commonly used, as the treatment window length $tw \rightarrow 0$. Here we demonstrate this convergence by fitting the model parameters with progressively smaller treatment window lengths. Table 5 lists the best-fit parameter values for the exponential model to the patient 5 data for treatment window lengths of 30, 15, 10, and 5 non-dimensional minutes, as well as $tw = dt$, where *dt* is the numeric integration step size of *fde12* (here we use either 1/288 or 0.001).

**Table 5:** Fitted parameter values for the exponential model with the continuous death-rate term, for patient 5 and various treatment window lengths, *tw*. Fitted parameter values for the exponential model with impulsively applied radiation cell-kill is included for comparison

*tw*	*a*	$\alpha$ (Gy^-1^)	SSR
1/48	0.011	0.022	8.112
1/96	0.012	0.022	8.115
1/144	0.012	0.024	8.118
1/288	0.012	0.022	8.127
$dt=0.001$	0.013	0.023	8.174
Impulse	0.0167	0.0243	8.397

As seen in Table 5, when using the death-rate radiation term, the fitted values of *a* and $\alpha$ are consistent, and the SSR values do not significantly vary. These fitted parameter values are close to those determined by fitting the impulsive model to the data. Of note, the goodness-of-fit metric is smaller for the models implementing the death-rate term than for the impulsive model.

### Radiation Death-Rate Term Gives Speed-Up of Computational Time

To demonstrate the increase in speed of computation using the continuous death-rate term instead of the Heaviside model or the most common, impulsive model, we run 100 parameter fitting instances on the patient 5 dataset. Using the exponential model as the baseline, comparison is made between the various implementations of the radiation-induced tumour reduction. That is, we compare the continuous death-rate term with hyperbolic tangent functions, ${\hat{H}}$, the discontinuous death-rate term with Heaviside functions, *H*, and the impulse model that stops and restarts the numeric integration at each treatment time. Note that since we are not considering fractional derivatives for this test, discontinuities are allowed, but do still pose numeric integration challenges.

In MATLAB (The Mathworks Inc 2021), we fit these models to the data $M=100$ times using *fmincon* and randomized uniformly sampled initial guesses for $a\in [0,0.2]$ and $\alpha \in [0,0.1]$. Both the total time (using *tic* and *toc*) and the total CPU time (using *cputime*) are recorded. The fitting procedure was run on a standard 2019 MacBook Pro laptop (1.4 GHz intel Core i5, 8 GB RAM). We divide the total time to complete all fits by *M* to get the average "time per fit" reported in Table 6. The models were numerically simulated with either *fde12*, a fixed time-step method ($dt=1/ {288}$) with no correction option, or *ode45*, a dynamic time-step method with maximum time-step set to $dt = 1 /{288}$.

**Table 6:** Comparison of computation time for the death-rate model with either a hyperbolic tangent ${\hat{H}}(t)$ function or a Heaviside *H*(*t*) function, and the impulsive (start/stop) model. Parameter fitting of the exponential growth model to patient 5 data was performed 100 times and the average total time and CPU time per fit are reported. Best-fitting parameters are also listed along with the frequency of occurrence (consensus)

	Model	Time per Fit	CPU time per Fit	Consensus	*a*	$\alpha$ (Gy^-1^)
*fde12*	Death-rate ${\hat{H}}(t)$	9.77 s	14.99 s	($SSR < 8.13$) 85/100	$a = 0.0118$	$\alpha = 0.0222$
	Death-rate *H*(*t*)	280.62 s	285.88 s	($SSR < 8.6$) 85/100	$a =0.0112$	$\alpha =0.0201$
	Impulsive Model	6.41 s	11.51 s	($SSR < 8.4$) 86/100	$a = 0.0167$	$\alpha = 0.0243$
*ode45*	Death-rate ${\hat{H}}(t)$	2.99 s	3.15 s	($SSR < 8.12$) 88/100	$a = 0.0115$	$\alpha = 0.0221$
	Death-rate *H*(*t*)	875.36 s	873.55 s	($SSR < 8.1$) 81/100	$a = 0.0114$	$\alpha = 0.0220$
	Impulsive Model	6.15 s	6.31 s	($SSR < 8.4$) 88/100	$a = 0.0167$	$\alpha = 0.0243$

As seen in Table 6, the fastest fits are obtained by the newly proposed continuous death-rate function solved via *ode45*. The standard impulsive model is almost twice as slow to find the best fit with this algorithm. Using *fde12*, the impulsive model is the fastest, but requires twice the CPU time as *ode45*. The discontinuous death-rate model implementing the Heaviside function is incredibly slow and not recommended.

The various death models do find approximately equal fits as measured by SSR, with approximately the same success rates $81-88$%. The fitted parameter values c*a* and $\alpha$ are slightly different for the different models but mostly invariant to the numeric solver.

The computing speed-up obtained by solving the ODE model with the continuous death-rate radiation term is considerable. Applications that rely on many repetitions of numeric integration of an ODE model, such as parameter fitting procedures and virtual clinical trial construction and simulations, will benefit greatly from this improvement.

## Discussion

This paper provided an in-depth comparison between ODE and FDE models of tumour growth with radiation treatment and determined that the added complexity of the fractional derivative does not significantly improve the model’s ability to simulate clinical data. Additionally, we proposed a new continuous radiation-induced death-rate function as a more biological and computationally efficient alternative to the impulsive model commonly used.

Upon fitting all 3 of our ODE and corresponding 3 FDE models to the data, we observe that in 86% of our FDE fits, the fractional order $\mu \in [0.999,1]$. The fitted curves for these fractional models are essentially identical to their respective ODE model predictions. Although our sensitivity analysis reveals the order of the fractional derivative to be the most sensitive of our parameters, fitted values of $\mu$ which do not approach 1 either don’t improve the fit of the model to the data or yield non-biological results. An examination of model selection information-based criterion finds, repeatedly, that the ODE exponential model is the best of our candidate models for this clinical dataset. The Akaike evidence ratios further substantiate our findings from the fits and in 93% of the patients show substantial evidence against the FDE model in favour of its ODE counterpart.

The continuous death-rate function describing radiation-induced cell kill post-treatment provides similar model predictions and data-fits to the impulsive model wherein the numeric integration is stopped and restarted after each treatment. Parameterizations and goodness-of-fit metrics are similar between the two models. The death-rate term provides a more biological description of cell death following radiation treatment as the treatment window length can be adjusted. Radiation induces DNA damage that, if not repaired, will cause the cell to die when it attempts to divide. Thus, the actual death following radiation treatment is spread out over the approximately 24-hour period when the cancer cells are attempting to proliferate. The radiation effect treatment window, *tw*, could be increased to try to capture this effect. In this work, we kept the treatment window short in order to aid comparison with the impulsive model, which assumes all death occurs instantaneously, but this assumption is not necessary.

Previous work applying FDE models to radiation therapy was proposed by Farayola et al. (2020a, 2020b) wherein they lumped the effect of all radiation fractions into one dose that was applied all together starting at $t=0$. This method of lumping the fractionated treatments together does not replicate the actual biology that occurs during tumour treatment, and it does not account for the tumour regrowth that occurs between treatments, an important dynamic of tumour treatment modelling (Enderling 2020). In comparison, the FDE models proposed here with the addition of the continuous death-rate term were all able to capture the dynamical features of tumour growth under fractionated radiation treatment.

The computational efficiency gained by the continuous death-rate term, over the impulsive model, is significant. Parameter fitting of ODE models is a time-consuming process, requiring many repetitions of numeric integration—especially for genetic algorithm, Markov Chain Monte Carlo, and simulated annealing-based methods. The continuity of the function describing the entire treatment course is also significant for gradient-based optimization methods that estimate derivatives to build Hessian matrices. The speed-up provided by this new death-rate expression will be noticeable for many applications in mathematical oncology and quantitative systems pharmacology.

## Data Availability

Data sharing was not applicable to this article as no datasets were generated directly during the current study.
